# A Galactic View of Nature's Decontamination Squad

**DOI:** 10.1371/journal.pbio.1001842

**Published:** 2014-04-22

**Authors:** Roland G. Roberts

**Affiliations:** Public Library of Science, Cambridge, United Kingdom

[Fig pbio-1001842-g001]While genome sequences now pour out of factories, each yielding in turn the sequences of thousands of proteins, it's been harder to scale up our understanding of what these proteins actually look like and what they do for a living. This is largely because DNA is DNA, wherever you look, enabling the same technology to be applied to any segment of any genome. Solving the three-dimensional molecular structure and biochemical function of any given protein, however, approaches uniqueness, involving labour-intensive X-ray crystallography or NMR and potentially open-ended functional studies.

**Figure pbio-1001842-g001:**
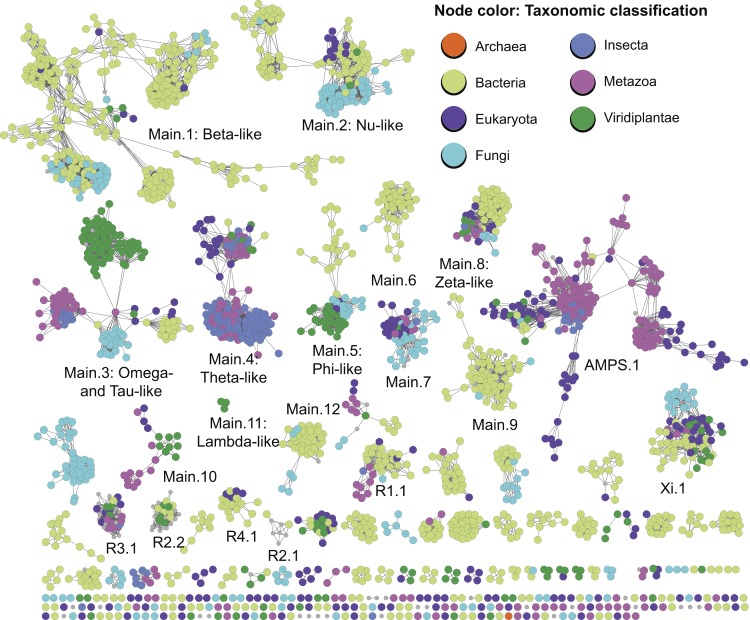
The cytGST night sky. Cytosolic glutathione S-transferases from across the tree of life.

In a paper just published in *PLOS Biology*, however, Susan Mashiyama, Patricia Babbitt, and colleagues point the way to one approach to bridging the informational chasm between sequence, structure, and function. As their model, they take a massive superfamily of proteins: the cytosolic glutathione S-transferases (cytGSTs). Glutathione is a main form of redox currency in living things, containing a central cysteine with a reactive sulfhydryl group that can be attached to a wide range of other molecules by nucleophilic attack; this is the classic reaction catalysed by cytGSTs. In some reactions, glutathione can also act as a reducing agent, resulting in its own oxidation to glutathione disulphide.

Some cytGSTs are known to perform intriguing and important functions, often using the attachment of hydrophilic glutathione to solubilise hydrophobic chemicals ready for detoxification. Many organisms reach for cytGSTs to help them cope with dietary toxins, pesticides, drugs, environmental poisons, and endogenous metabolites. This is a group of enzymes encoded by genomes across the tree of life, and a search for GSTs pulls out 30,000 studies in PubMed. However, our structural and functional knowledge of the superfamily is piecemeal and covers only a small and unrepresentative proportion of its members. The authors of the current study make a broad and systematic survey of the state of our knowledge of cytGSTs, and then start an equally systematic attempt to fill in the gaps.

They kick off by compiling a nonredundant catalogue of more than 13,000 cytGST sequences, using them to construct a network of sequence and structural similarity relationships. The resulting tangled web is so complex that it can't be visualised on conventional computers, so the authors treat us to a number of representative pared-down snapshots to illustrate varying degrees of granularity of sequence space. They then decorate the network with information gleaned from the literature, including structure, chemical reaction type, and the phylogenetic representation of these proteins across the biosphere. The cytGST network is strikingly reminiscent of the night sky, with the initial impression of a handful of obvious constellations giving way to the realisation of a dizzying underlying complexity.

Most sobering is the overwhelming paucity of our knowledge of even this intensively studied superfamily—the network falls naturally into many very distinct families, some identified here for the first time (35 distinct novel subgroups and over a hundred singletons), and most with little or no information beyond the protein sequence. Like the universe, protein sequence space is dominated by dark matter.

The authors then use this new global overview of cytGST sequence space to prioritise a more rational survey of the diverse structures and functions of superfamily members. Widely distributed representatives of poorly characterised branches of the network are targeted for crystallisation, an endeavour that delivers 37 new molecular structures of 27 proteins. In parallel, they also use a high-throughput assay system to test similarly representative cytGSTs for their ability to catalyse 20 known GST reactions. This confirms GST activity in 82 diverse cytGSTs, increasing by 50% the number of enzymes for which this has been demonstrated experimentally.

Like a genome sequence, the broad framework of protein sequence space presented here, with its preliminary structural and functional overlay, represents a beginning and a means to an end, rather than an end in itself. Details of the new structures will be described elsewhere, as will the kinetic properties of the enzymes. The authors do tantalise us with a taste of things to come, scrutinising the sequence-structure-function relationships of diverse cytGSTs that catalyse disulphide bond reduction (one of the reactions where two molecules of glutathione are oxidised to glutathione disulphide), and using their network to identify candidate cytGSTs for bioremediation of chlorinated organic pollutants. But the principal point of the current study is to function as an overarching framework for the substantial work that lies ahead, as these authors and others will presumably now try to fill in more of the missing information for this superfamily. And presumably other superfamilies would benefit from the same cosmological treatment.

**Mashiyama ST, Malabanan MM, Akiva E, Bhosle R, Branch MC, et al. (2014) Large-Scale Determination of Sequence, Structure, and Function Relationships in Cytosolic Glutathione Transferases across the Biosphere.** doi:10.1371/journal.pbio.1001843

